# The value of preoperative neutrophil‐to‐lymphocyte ratio, platelet‐to‐lymphocyte ratio, and red blood cell distribution width in predicting positive surgical margin after laparoscopic radical prostatectomy

**DOI:** 10.1002/cnr2.1977

**Published:** 2024-01-23

**Authors:** Hao Wang, Dawei Xie, Siqi Wang, Liyang Wu, Yifan Chu, Pushen Yang, Weifeng He, Jianwen Wang

**Affiliations:** ^1^ Department of Urology Beijing Chaoyang Hospital, Capital Medical University Beijing China; ^2^ Department of Urology Capital Medical University Daxing Teaching Hospital Beijing China

**Keywords:** neutrophil‐to‐lymphocyte ratio, platelet‐to‐lymphocyte ratio, positive surgical margin, prostate cancer, red blood cell distribution width

## Abstract

**Background:**

Prostate cancer (PCa) is one of the most common malignant tumors in men, and laparoscopic radical prostatectomy (LRP) is commonly used to treat localized and advanced PCa. Positive surgical margin (PSM) is one of the most frequent problems faced by surgeons.

**Aims:**

This study aimed to explore the value of the neutrophil‐to‐lymphocyte ratio (NLR), platelet‐to‐lymphocyte ratio (PLR), and red blood cell distribution width (RDW) in predicting PSM after LRP.

**Methods and Results:**

Three hundred and twenty patients with PCa were admitted and underwent LRP in Beijing Chaoyang Hospital from January 2017 to June 2023. Patients were randomly divided into a training set (225 cases) and a validation set (95 cases) in a 7:3 ratio. NLR, PLR, and RDW were significantly higher in the PSM group than in the negative surgical margins (NSM) group. In addition, the NLR, PLR, and RDW values correlated with clinical *T* stage, Gleason score, and seminal vesicle invasion in the PSM group. In training set, ROC curve analysis revealed that the optimal cutoff values of NLR, PLR, and RDW for predicting postoperative PSM in PCa were 2.31, 115.40, and 12.85%, respectively. Multivariate Logistic regression analysis showed NLR and RDW were the clinical independent predictors. The area under the curve (AUC, 0.770, 95% CI 0.709–0.831) for postoperative PSM was the highest when a combination of the three parameters was used, with sensitivity and specificity of 62.5% and 85.2%, respectively. In validation set, the AUC values for NLR, PLR, RDW and the three markers combined were 0.708, 0.675, 0.723, and 0.780, respectively. Correlation analysis showed that in the PSM group, NLR was positively correlated with PLR and RDW, and PLR was positively correlated with RDW. By contrast, in the NSM group, a positive association was only found between NLR and PLR.

**Conclusions:**

Higher preoperative NLR, PLR, and RDW values were associated with postoperative PSM. Additionally, the three markers combined may be useful to predict PSM.

## 1 INTRODUCTION

Prostate cancer (PCa) is one of the most common malignant tumors of the urogenital system in middle‐aged and elderly men, posing a serious risk to psychological and physical well‐being.^1^ For patients with localized and advanced PCa, radical prostatectomy (RP)—either laparoscopic (LRP) or robot‐assisted (RARP)—is one of the most established therapies, yielding a longer life expectancy.^2^ Positive surgical margin (PSM) is one of the most frequent problems faced by surgeons. PSM depends on various factors, including prostate anatomy, tumor features (size, stage, and localization), and the surgical technique used.^3^ PSM is considered an adverse pathological feature that may lead to biochemical recurrence (BCR), metastasis, and cancer‐specific death, exposing patients to the risk of further treatments, such as adjuvant or salvage radiotherapy with or without androgen blockade.^4^, ^5^ Zhang et al. demonstrated that PSM is significantly associated with an increased risk of BCR and may serve as an independent prognostic factor in PCa patients.^6^ Based on the worse oncologic outcomes associated with PSM, preoperative judgment of the surgical margin is important to reduce PSM rates. Recently, prediction models based on clinicopathological features and imaging results, such as nomograms, have been established for clinical convenience.^7^, ^8^, ^9^

Cancer is widely thought to be caused by chronic inflammation.^10^ Several inflammatory markers, such as neutrophil‐to‐lymphocyte ratio (NLR) and platelet‐to‐lymphocyte ratio (PLR), comprehensively reflect inflammation and immune status in patients with various cancers.^11^, ^12^ The impact of NLR, PLR and other blood‐derived metrics has been extensively studied across urological malignancies from the prognostic point of view.^13^, ^14^ Another parameter, red blood cell distribution width (RDW), reflects the heterogeneity of peripheral blood erythrocyte volume and is primarily used to diagnose various clinical anemias. RDW is also linked to long‐term inflammation and poor health, while abnormal inflammation and nutritional status may be risk factors for carcinoma development.^15^ The higher the tumor stage, the more local or systemic inflammatory responses are promoted, leading to increased RDW values.^16^ Albayrak et al. showed that RDW was increased in PCa patients and was significantly associated with disease progression.^17^

To date, the predictive value of preoperative NLR, PLR, and RDW levels for postoperative PSM in PCa patients has not been assessed. To address this research gap, in this study, we collected clinical and pathological characteristics as well as preoperative NLR, PLR, and RDW values for PCa patients who underwent LRP. We investigated the capacity of these parameters to predict PSM, with the aim of providing information that could be of potential benefit to urological surgeons carrying out this procedure in the future.

## 2 MATERIALS AND METHODS

### 2.1 Patients

We conducted a retrospective study on PCa patients who underwent LRP at Beijing Chaoyang Hospital from January 2017 to June 2023. The inclusion criteria were as follows: (1) PCa was diagnosed by ultrasound‐guided prostate biopsy and postoperative pathological results; (2) peripheral blood had been collected before LRP, with corresponding results provided; (3) patients had not received preoperative neoadjuvant endocrine therapy, radiotherapy, or chemotherapy; (4) the patients' clinicopathological data were complete, including previous hospitalization medical records, laboratory examination reports, and pathological diagnosis reports. The exclusion criteria were as follows: (1) patients suffering from other malignant tumors, acute and chronic infections, or autoimmune system diseases; (2) patients with severe heart, liver, or kidney diseases; (3) patients taking immunosuppressive drugs or suffering from hematologic diseases that might affect the results of routine blood tests. After these exclusions, 320 patients were enrolled in the study. Patients were randomly divided into a training set (225 cases) and a validation set (95 cases) in a 7∶3 ratio. Then, the patients were divided into PSM and negative surgical margin (NSM) groups according to post‐operation pathology. The study was conducted in accordance with the Declaration of Helsinki and was approved by the ethics committee of Beijing Chaoyang Hospital, China.

### 2.2 Data collection

Baseline characteristics and clinicopathological data were obtained from the medical records database. Clinical data mainly included age, weight, preoperative maximum serum prostate‐specific antigen (PSA), prostate volume (PV), and clinical stage. Body mass index (BMI) values were classified by applying the World Health Organization criteria. PV was obtained by preoperative ultrasound or magnetic resonance imaging (MRI). f/t PSA was calculated as the ratio of free PSA to total PSA, and PSA density (PSAD) was determined by calculating the ratio of PSA to prostate volume. Clinical stage was derived based on the evaluation of the patients' clinical data, using the 8th edition of the American Joint Committee on Cancer staging system. Preoperative Gleason score (GS) and biopsy positive core (BPC) ratio were derived from preoperative biopsy pathology. Using the D'Amico classification, low‐risk PCa was defined as PSA <10.0 ng/mL, GS <7, and clinical stage T1c to T2a at initial 10‐core biopsy. Intermediate‐risk PCa was defined as PSA ≥10 ng/mL and <20 ng/mL, GS of 7, and clinical stage T2b. High‐risk PCa patients were PSA >20.0 ng/mL, GS >7, and clinical stage ≥ T2c. The involvement of surgical margin positivity, seminal vesicle invasion, capsule invasion, and lymph nodes was determined by postoperative pathology. PSM was confirmed if, on evaluation of postoperative pathology by at least two pathologists, tumor cells were visible microscopically at the surgical margins of tumors.

Venous blood (2 mL) was collected from patients within a week before surgery (in the morning) and placed in EDTA anticoagulation tubes and drying tubes. The samples were sent to the laboratory department where a hematology analyzer was used to obtain absolute neutrophil counts, absolute lymphocyte counts, blood platelet counts, and RDW (directly). NLR and PLR values were obtained according to the following formulae: NLR = absolute neutrophil count/lymphocyte count; PLR = blood platelet count/lymphocyte count.

### 2.3 Statistical analysis

Statistical analysis was performed using SPSS 26.0 (IBM Corp., Armonk, NY) and GraphPad Prism 9.5 (GraphPad Software, San Diego, CA). The independent *t*‐test and χ^2^ test were used to compare between‐group differences. Multivariate Logistic regression analysis was used to determine the clinical independent predictors. Area under the curve (AUC), used as a summary measure of the receiver operating characteristics (ROC) curve, represents discrimination ability. AUC is expressed on a scale of 0.5 to 1 (the larger the AUC value, the better the classification effect). Sensitivity and specificity were defined using ROC curves, and differences in AUC values were analyzed using GraphPad Prism 9.5. Correlations among NLR, PLR, and RDW were analyzed using Spearman's correlation: r >0 indicates a positive correlation between two variables, while r < 0 indicates a negative correlation. 0 < r < 1 indicates a certain degree of linear correlation between the two variables, as follows: r < 0.4 is a low linear correlation, 0.4 ≤ r < 0.7 is a significant correlation, and 0.7 ≤ r < 1 is a high linear correlation. Results were reported as numbers (*n*) and percentages (%), means and standard deviations, or AUC with 95% confidence intervals (CI), as appropriate, and were considered statistically significant at a *p*‐value <.05 in two‐tailed tests.

## 3 RESULTS

### 3.1 Baseline characteristics

A total of 320 PCa patients who had undergone LRP were enrolled in this study. In training set, patients were divided into two groups based on surgical margin status obtained from pathological results: negative surgical margin (NSM, 81/225, 36%) and PSM (144/225, 64%). The number of NSM and PSM were 35 (36.8%) and 60 (63.2%) in validation set. Comparisons of the clinical and pathological data are shown in Table 1, there is no significant difference between training and validation sets. In Table 2, we performed univariate analysis of risk factors for surgical margins between NSM and PSM groups in training set. No significant differences in age, BMI, prostate volume, preoperative maximum PSA, f/t PSA, or PSAD were noted between the two groups (*P* > .05). The proportions of patients with low, intermediate, and high D'Amico risk classification in the NSM group were 23.5%, 35.8%, and 40.7%, respectively, while they were 11.1%, 30.6%, and 58.3% in the PSM group; these differences were significant (*P =* .014). Among the perioperative pathology variables, in the PSM group, BPC ratio (0.51 ± 0.28 vs. 0.41 ± 0.25, *P =* .008), biopsy GS (≤6, 7, ≥ 8: 16.7%, 34.7%, 48.6% vs. 29.6%, 38.2%, 32.2%, *P =* .023), and clinical T stage (T2, T3 ~ 4: 65.4%, 34.6% vs. 54.9%, 45.1%, *P* = .009) were significantly higher than in the NSM group. As shown in Table 2, in terms of postoperative characteristics, there were significant differences in pathological GS (≤6, 7, ≥ 8: 14.8%, 58.0%, 27.2% vs. 7.6%, 50.7%, 41.7%, *P =* .047), seminal vesicle invasion (16.0% vs. 30.6%, *P* = .016), and pelvic lymph node involvement (3.7% vs. 10.4%, *P* = .048) between patients in the two groups. However, there was no significant difference in prostate capsule invasion (18.5% vs. 17.4%, *P =* .827). In addition, we performed the same analysis on validation set, and the results were similar (Supplementary Table S1).

**TABLE 1:** Clinical and pathological characteristics of patients received LRP between training and validation sets.

Characteristics	Training set (*n* = 225)	Validation set (*n* = 95)	t/χ^2^	*P* value
Age, years	68.25 ± 6.58	68.39 ± 6.80	0.167	.867
BMI, kg/m^2^	25.08 ± 3.00	25.22 ± 3.13	0.368	.713
Preoperative maximum PSA, ng/mL	19.81 ± 20.73	16.17 ± 15.65	1.536	.125
PV, cm^3^	37.92 ± 18.05	38.05 ± 16.73	0.061	.951
f/t PSA	0.13 ± 0.08	0.13 ± 0.07	0.233	.816
PSAD, ng/mL^2^	0.58 ± 0.58	0.48 ± 0.49	1.557	.120
BPC ratio	0.47 ± 0.27	0.44 ± 0.24	1.079	.282
NLR	2.23 ± 0.79	2.09 ± 0.77	1.476	.141
PLR	123.12 ± 41.25	113.99 ± 38.85	1.840	.067
RDW (%)	12.85 ± 0.88	12.89 ± 0.81	0.362	.718
cT stage			0.561	.454
T2	132	60		
T3 ~ T4	93	35		
Biopsy GS			1.108	.575
≤6	48	25		
7	81	30		
≥8	96	40		
Pathological GS			0.812	.666
≤6	23	7		
7	120	50		
≥8	82	38		

Abbreviations: BMI, body mass index; BPC, biopsy positive cores; cT stage, clinical T stage; GS, Gleason score; LRP, laparoscopic radical prostatectomy; NLR, neutrophil to lymphoctye ratio; PLR, platelte to lymphocyte ratio; PSA, prostate specific antigen; PSAD, prostate specific antigen density; PV, prostate volume; RDW, red blood cell distribution width.

**TABLE 2:** Univariate analysis of risk factors for surgical margins between NSM and PSM groups in training set.

Characteristics	*n* = 225 (%)	NSM group (*n* = 81)	PSM group (*n* = 144)	t/χ^2^	*P* value
Age (*n*, %), years				1.661	.197
≤65	74 (32.9)	31 (38.3)	43 (29.9)		
>65	151 (67.1)	50 (61.7)	101 (70.1)		
BMI (*n*, %), kg/m^2^				0.004	.952
BMI<24.0	70 (31.1)	25 (30.9)	45 (31.3)		
BMI≥24.0	155 (68.9)	56 (69.1)	99 (68.7)		
Preoperative maximum PSA (*n*,%), ng/mL				3.281	.194
<10	80 (35.6)	35 (43.2)	45 (31.3)		
10 ~ 20	87 (38.7)	27 (33.3)	60 (41.7)		
>20	58 (25.7)	19 (23.5)	39 (27.0)		
PV (*n*, %), cm^3^				0.909	.635
<40	139 (62.3)	51 (63.0)	88 (61.1)		
40 ~ 70	70 (30.5)	26 (32.1)	44 (30.6)		
>70	16 (7.3)	4 (4.9)	12 (8.3)		
f/t PSA ($\bar{x}$ ± SD)	225 (100)	0.12 ± 0.06	0.13 ± 0.09	1.064	.228
PSAD ($\bar{x}$ ± SD), ng/mL^2^	225 (100)	0.60 ± 0.70	0.58 ± 0.52	0.250	.803
BPC ratio ($\bar{x}$ ± SD)	225 (100)	0.41 ± 0.25	0.51 ± 0.28	2.695	.008*
NLR	95 (100)	1.85 ± 0.58	2.44 ± 0.81	5.719	.000*
PLR	95 (100)	108.48 ± 33.21	131.36 ± 43.11	4.135	.000*
RDW (%)	95 (100)	12.45 ± 0.70	13.07 ± 0.91	5.314	.000*
cT stage (*n*, %)				6.732	.009*
T2	132 (58.7)	53 (65.4)	79 (54.9)		
T3 ~ T4	93 (41.3)	28 (34.6)	65 (45.1)		
D'Amico classification (*n*, %)				8.605	.014*
Low	35 (15.6)	19 (23.5)	16 (11.1)		
Intermediate	73 (32.4)	29 (35.8)	44 (30.6)		
High	117 (52.0)	33 (40.7)	84 (58.3)		
Biopsy GS (*n*, %)				7.578	.023*
≤6	48 (21.4)	24 (29.6)	24 (16.7)		
7	81 (36.0)	31 (38.2)	50 (34.7)		
≥8	96 (42.6)	26 (32.2)	70 (48.6)		
Pathological GS (*n*, %)				6.127	.047*
≤6	23 (10.2)	12 (14.8)	11 (7.6)		
7	120 (53.3)	47 (58.0)	73 (50.7)		
≥8	82 (36.5)	22 (27.2)	60 (41.7)		
Prostate capsule invasion (*n*, %)				0.048	.827
Yes	40 (17.8)	15 (18.5)	25 (17.4)		
No	185 (82.2)	66 (81.5)	119 (82.6)		
Seminal vesicle invasion (*n*, %)				5.767	.016*
Yes	57 (25.3)	13 (16.0)	44 (30.6)		
No	168 (74.7)	68 (84.0)	100 (69.4)		
Pelvic lymph nodes involvement (*n*, %)				3.927	.048*
Yes	18 (8.0)	3 (3.7)	15 (10.4)		
No	107 (47.6)	44 (54.3)	63 (43.8)		

Abbreviations: BMI, body mass index; BPC, biopsy positive cores; cT stage, clinical T stage; GS, Gleason score; NLR, neutrophil to lymphoctye ratio; NSM, negative surgical margins; PLR, platelte to lymphocyte ratio; PSA, prostate specific antigen; PSAD, prostate specific antigen density; PSM, positive surgical margins; PV, prostate volume; RDW, red blood cell distribution width.

* Means the *p*‐value<.05 is considered statistically significant.

### 3.2 Comparison of serum levels of NLR, PLR, and RDW between NSM and PSM groups

NLR, PLR, and RDW values were significantly higher in the PSM group than in the NSM group (Table 2): NLR, 2.44 ± 0.81 versus 1.85 ± 0.81 (*P* < .001); PLR, 131.36 ± 43.11 versus 108.48 ± 33.21 (*P* < .001); RDW, 13.07 ± 0.91% versus 12.45 ± 0.70% (*P* < .001), respectively. As shown in Table 3, multivariate Logistic regression analysis showed NLR (odds ratio, OR = 2.896, 95% CI 1.418 ~ 5.916, *P* < .05) and RDW (OR = 2.509, 95% CI 1.590 ~ 3.960, *P* < .001) were the clinical independent predictors.

**TABLE 3:** Multivariate Logistic regression analysis of independent risk factors affecting surgical margins in training set.

Factors	B	SE	Wald	*P*	OR	95% CI
BPC ratio	0.010	0.008	1.489	.222	1.010	0.994 ~ 1.025
NLR	1.063	0.364	8.515	.004	2.896	1.418 ~ 5.916
PLR	0.006	0.006	1.065	.302	1.006	0.994 ~ 1.018
RDW	0.920	0.233	15.636	.000	2.509	1.590 ~ 3.960
cT stage	0.130	0.571	0.052	.820	1.139	0.372 ~ 3.488
D'Amico classification						
Low					1.000	
Intermediate	0.669	0.625	1.147	.284	1.953	0.754 ~ 6.647
High	0.862	0.813	1.123	.289	2.367	0.481 ~ 11.650
Biopsy GS						
≤6					1.000	
7	−0.650	0.597	1.184	.277	0.522	0.162 ~ 1.684
≥8	−0.990	0.809	1.497	.211	0.376	0.076 ~ 1.814
Pathological GS						
≤6					1.000	
7	0.060	0.544	0.012	.911	1.062	0.366 ~ 3.083
≥8	−0.581	0.677	0.737	.391	0.559	0.148 ~ 2.108
Prostate capsule invasion	−0.562	0.574	0.985	.328	0.570	0.185 ~ 1.757
Seminal vesicle invasion	0.185	0.604	0.094	.760	1.203	0.368 ~ 3.930

Abbreviations: BPC, biopsy positive cores; CI, confidence interval; cT stage, clinical T stage; GS, Gleason score; NLR, neutrophil to lymphoctye ratio; OR, odds ratio; PLR, platelte to lymphocyte ratio; RDW, red blood cell distribution width.

### 3.3 Relationship between NLR, PLR, and RDW levels and clinicopathological characteristics in the PSM group

We analyzed the 144 patients within the PSM subgroup in training set (Table 4). Among the clinicopathological variables, there were no significant associations between NLR, PLR, or RDW and age, BMI, or pelvic lymph node involvement (*P* > .05). However, NLR, PLR, and RDW levels were significantly associated with higher biopsy pathological GS, cT stage, and seminal vesicle invasion (*P* < .05) in the PSM groups. NLR values were significantly higher in patients with prostate capsule invasion (2.82 ± 0.69 vs. 2.36 ± 0.82, *P* = .009), while PLR (144.14 ± 44.22 vs. 128.67 ± 42.57, *P* = .103) and RDW (13.02 ± 0.84% vs. 13.08 ± 0.92%, *P* = .786) levels were not significantly different in these patients (Table 4).

**TABLE 4:** Relationship between NLR, PLR, RDW levels and clinicopathological characteristics in PSM group of training set.

Characteristics	*n* = 144 (%)	NLR	*t*	*P* value	PLR	*t*	*P* value	RDW (%)	*t*	*P* value
Age, years			1.166	.246		0.163	.871		0.495	.621
≤65	43 (29.9)	2.31 ± 0.79			132.25 ± 32.08			13.01 ± 0.76		
>65	101 (70.1)	2.49 ± 0.82			130.97 ± 47.17			13.09 ± 0.96		
BMI, kg/m^2^			0.211	.833		1.016	.312		0.260	.795
BMI<24.0	45 (31.3)	2.42 ± 0.84			125.95 ± 48.47			13.10 ± 1.00		
BMI≥24.0	99 (68.7)	2.45 ± 0.81			133.82 ± 40.46			13.06 ± 0.86		
cT stage			5.070	.000*		3.071	.003*		2.503	.013*
T2	79 (54.9)	2.15 ± 0.76			121.63 ± 43.59			12.90 ± 0.82		
T3 ~ T4	65 (45.1)	2.79 ± 0.73			143.18 ± 39.73			13.27 ± 0.96		
Biopsy GS			15.317	.000*		5.240	.000*		3.163	.002*
≤7	74 (51.4)	1.82 ± 0.44			114.54 ± 28.76			12.85 ± 0.93		
>7	70 (48.6)	3.10 ± 0.56			149.13 ± 48.50			13.31 ± 0.81		
Pathological GS			11.200	.000*		4.469	.000*		5.072	.000*
≤7	84 (58.3)	1.97 ± 0.57			118.61 ± 30.21			12.77 ± 0.75		
>7	60 (41.7)	3.10 ± 0.62			149.21 ± 51.62			13.49 ± 0.94		
Prostate capsule invasion			2.636	.009*		1.641	.103		0.272	.786
Yes	25 (17.4)	2.82 ± 0.69			144.14 ± 44.22			13.02 ± 0.84		
No	119 (82.6)	2.36 ± 0.82			128.67 ± 42.57			13.08 ± 0.92		
Seminal vesicle invasion			5.998	.000*		3.537	.001*		2.788	.006*
Yes	44 (30.6)	2.99 ± 0.71			149.78 ± 44.37			13.38 ± 1.04		
No	100 (69.4)	2.20 ± 0.74			123.25 ± 40.15			12.93 ± 0.80		
Pelvic lymph nodes involvement			1.608	.112		1.790	.077		0.375	.709
Yes	15 (10.4)	2.77 ± 0.81			154.19 ± 54.02			12.93 ± 0.94		
No	63 (43.8)	2.43 ± 0.71			131.23 ± 42.23			13.01 ± 0.78		

Abbreviations: BMI, body mass index; cT stage, clinical T stage; GS, Gleason score; NLR, neutrophil to lymphoctye ratio; PLR, platelte to lymphocyte ratio; PSM, positive surgical margins; RDW, red blood cell distribution width.

* Means the *p*‐value<.05 is considered statistically significant.

### 3.4 Capacity of preoperative NLR, PLR, and RDW levels to predict postoperative PSM in PCa patients

**FIGURE 1:**
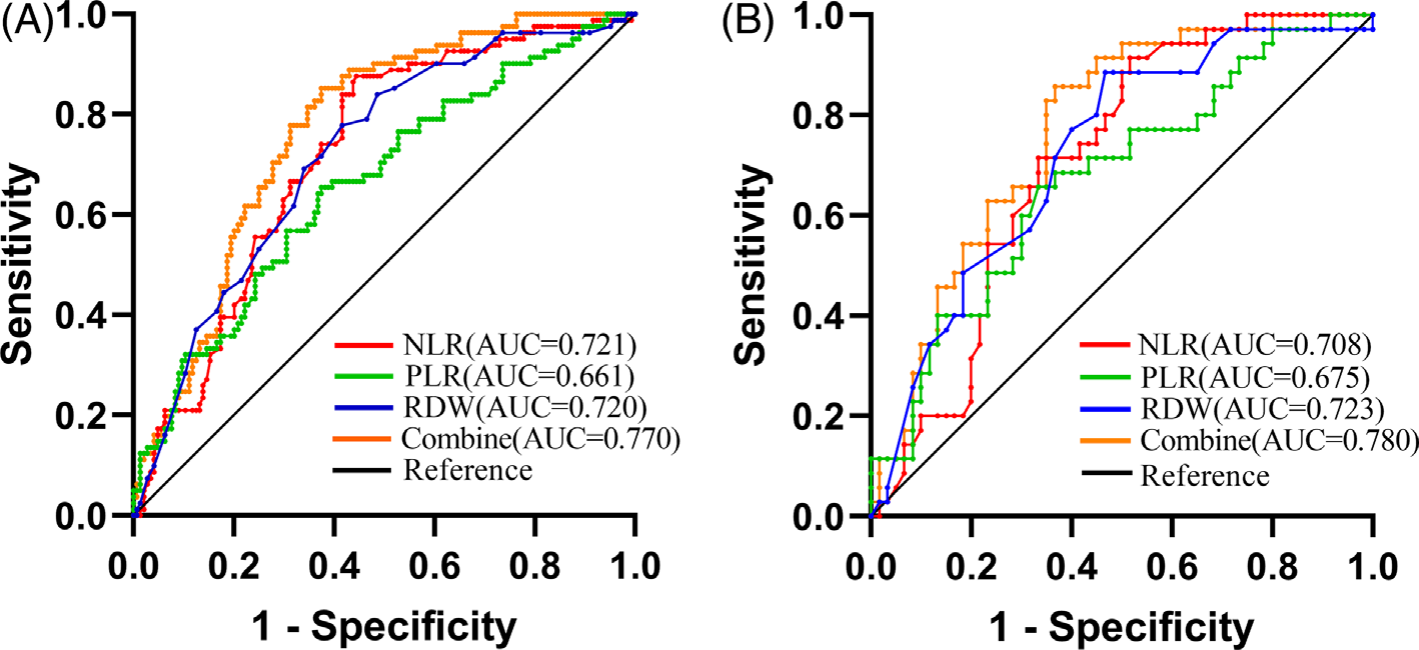
ROC curve of preoperative NLR, PLR, and RDW levels predicting postoperative PSM of PCa patients in training (A) and validation (B) sets.

In training set, the optimal cutoff values for NLR, PLR, and RDW levels to predict postoperative PSM in PCa patients after LRP were 2.31, 115.40, and 12.85%, respectively (Table 5). Among the three biomarkers, PLR predicted PSM and NSM with the highest sensitivity (62.5%), while NLR and RDW were more specific (87.7% and 77.8%, respectively). ROC analysis was then used to assess the AUCs for the single and combined biomarkers. For NLR, PLR, and RDW, the AUC values were 0.721 (95% CI 0.654 ~ 0.788, *P* < .001), 0.661 (95% CI 0.587 ~ 0.735, *P* < .001), and 0.720 (95% CI 0.682 ~ 0.788, *P* < .001), respectively. In addition, combining the three biomarkers to predict PSM yielded an AUC of 0.770 (95% CI 0.709 ~ 0.831, *P* < .001), which was higher than the AUC values obtained for NLR, PLR, and RDW as individual markers. The sensitivity and specificity of the combined biomarkers were 62.5% and 85.2%, respectively (Table 5 and Figure 1A). As shown in Table 5, we used validation set for confirming the cutoff values determined using the training set to evaluate sensitivity and specificity, the AUC values for NLR, PLR, RDW and the three markers combined in validation set were 0.708 (95% CI 0.606 ~ 0.811, *P* < .05), 0.675 (95% CI 0.563 ~ 0.787, *P* < .05), 0.723 (95% CI 0.619 ~ 0.827, *P* < .001) and 0.780 (95% CI 0.677 ~ 0.864, *P* < .001), respectively (Table 5 and Figure 1B). The findings suggested that the training and validation sets about the NLR, PLR, and RDW had comparable predictive capabilities.

**TABLE 5:** Value of preoperative NLR, PLR, and RDW levels predicting postoperative PSM in training and validation set.

Index	Optimal cutoff value	AUC	AUC 95% CI	Sensitivity (%)	Specificity (%)	Youden Index	*P* value
NLR							
Training set	2.31	0.721	0.654 ~ 0.788	55.6	87.7	0.433	.000*
Validation set		0.708	0.606 ~ 0.811	48.3	91.4	0.397	.001*
PLR							
Training set	115.40	0.661	0.587 ~ 0.735	62.5	65.4	0.279	.000*
Validation set		0.675	0.563 ~ 0.787	46.7	77.1	0.238	.005*
RDW							
Training set	12.85	0.720	0.682 ~ 0.788	58.3	77.8	0.361	.000*
Validation set		0.723	0.619 ~ 0.827	60.0	77.1	0.371	.000*
Three combination							
Training set	—	0.770	0.709 ~ 0.831	62.5	85.2	0.477	.000*
Validation set	—	0.780	0.677 ~ 0.864	63.3	85.7	0.490	.000*

Abbreviations: AUC, area under the curve; CI, confidence interval; NLR, neutrophil to lymphoctye ratio; PLR, platelte to lymphocyte ratio; PSM, positive surgical margins; RDW, red blood cell distribution width.

* Means the *P*‐value<.05 is considered statistically significant.

### 3.5 Correlation between NLR, PLR, and RDW values in the PSM and NSM groups

**FIGURE 2:**
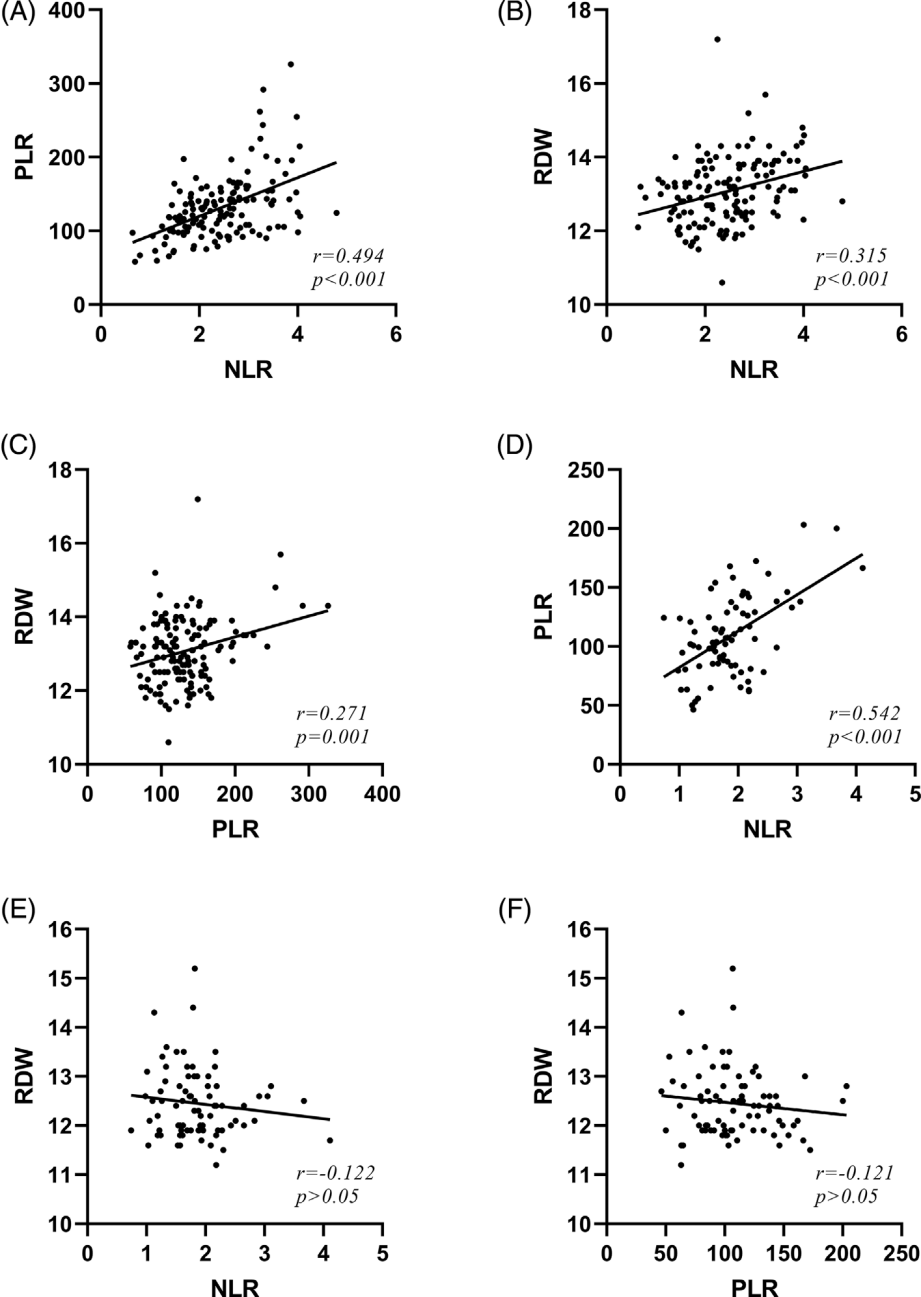
The correlation between NLR, PLR, and RDW levels of PSM (A, B, C) and NSM (D, E, F) groups.s.

Correlation analysis showed that in the PSM group, NLR was positively correlated with PLR (*r* = 0.494, *P* < .001) and RDW (*r* = 0.315, *P* < .001), and RDW was also positively correlated with PLR (*r* = 0.271, *P* = .001) (Figure 2A–C). Comparison of the correlation coefficients showed that the correlation between NLR and PLR was stronger than the correlation between RDW and either NLR or PLR. We also assessed the relationship between NLR, PLR, and RDW in the NSM group. The results showed that there was no significant correlation between RDW and either NLR (*r* = −0.122, *P* > .05) or PLR (*r* = −0.121, *P* > .05), although we did find a significant correlation between NLR and PLR (*r* = 0.542, *P* < .001) (Figure 2D–F).

## 4 DISCUSSION

RP is the standard first‐line treatment modality for patients with localized PCa, particularly in intermediate and high‐risk patients. In our study, the incidence of PSM was 64%, which is considerably higher than the previously reported figures of 10% to 35% among patients who have undergone RP.^4^, ^18^ This higher incidence may be related to the higher proportion of intermediate and high‐risk patients in our study (264/320, 82.50%). To date, multiple independent predictors of PSM have been identified, including preoperative PSA, biopsy and pathological GS, percentage of positive biopsy needles, nerve infiltration, pathological stage, lymph node positivity, extracapsular extension of the tumor, and seminal vesicle infiltration.^19^, ^20^, ^21^, ^22^ Consistent with these findings, the results of the current study revealed that the PSM and NSM groups showed significant differences in BPC ratio, clinical *T* stage, D'Amico classification, GS, seminal vesicle invasion, and pelvic lymph node positivity. However, there were no significant differences in capsule invasion between the two groups, which could possibly be attributed to sample size limitations, as well as individual differences.

In our study, NLR, PLR, and RDW were significantly higher in the PSM group than in the NSM group and NLR and RDW were the independent predictors for PSM. Systemic inflammation is linked to tumor development and patient outcomes and is thought to be a feature of tumorigenesis and progression.^23^, ^24^ Inflammation is also associated with obesity and metabolic diseases.^25^ The majority of patients in this study were overweight (217/320, 67.8%), which indicates these patients may have been in a state of chronic inflammation. Inflammation can promote the migration of neutrophils to peritumor tissues, which then release reactive oxygen species, causing oxidative damage to DNA cells, and secrete large amounts of vascular endothelial growth factors. In turn, these events induce chemotactic factor secretion by endothelial cells, leading to changes in vascular permeability, and stimulate tumor cell proliferation, as well as inhibiting the lymphocyte‐mediated anti‐tumor immune response.^26^, ^27^, ^28^ Tumor cells also produce cancer‐associated inflammatory factors that promote neutrophil expansion.^29^ Platelet abnormalities and malignancy have been linked in both basic and clinical studies, while reactive thrombocytosis is common in solid tumors.^30^, ^31^ Platelet activation promotes tumor angiogenesis, extracellular matrix degradation, and the release of adhesion molecules and growth factors and boosts tumor growth and metastasis.^32^, ^33^ In addition, lymphocytes play an important role in tumor cell destruction and apoptosis and can activate anti‐tumor immune factors directly or indirectly, thus inhibiting tumor metastasis and recurrence. Mantovani demonstrated that large numbers of neutrophils suppress the activation and antitumor activity of lymphocytes and natural killer cells.^34^ Furthermore, dependent on patient age and nutritional status, along with tumor progression, the immune system can become suppressed or weakened, resulting in reduced lymphocyte numbers. In summary, the inflammatory response is characterized by increased neutrophil levels and decreased lymphocyte levels. Thus, NLR and PLR reflect the level of systemic inflammation and the balance of the immune response, serving as potential biomarkers to characterize individual tumors in terms of angiogenesis, progression, and metastasis. In addition, RDW (which can be determined directly from blood tests) reflects the heterogeneity of red cell volume. Inflammatory cytokines have been shown to inhibit the stimulatory effects of erythropoietin on bone marrow erythrocyte stem cells, including the induction of cell maturation and suppression of apoptosis. Thus, inflammation causes more immature red blood cells to be released into the peripheral blood circulation, increasing the heterogeneity of peripheral red blood cells and leading to higher RDW values.^35^ Patients with advanced cancers, including PCa, are also often in a state of malnutrition, which may result in deficiencies in iron, vitamin B12, and folic acid, and varying degrees of anemia, as well as increased RDW.^16^

Subgroup analysis within the PSM group showed that increased NLR, PLR, and RDW were associated with clinical *T* stage, biopsy and pathological GS, capsule infiltration, and seminal vesicle invasion. The reason for these findings may be that patients with advanced carcinoma have faster disease progression and exhibit stronger inflammatory responses within the tumor tissue, resulting in abnormally high NLR, PLR, and RDW values. Our results are consistent with those of Rulando et al.,^36^ who showed that NLR was associated with higher GS (*r* = 0.572, *P* = .001). Gokce et al. also reported that high NLR was associated with higher GS, higher progression rates, and poorer prognosis.^37^ For PLR, Neofytou et al. showed that elevated PLR was related to poor tumor stage, pathological *T* stage, and degree of differentiation.^38^ Meanwhile, Huang et al. demonstrated that RDW was an independent risk factor for clinically significant PCa,^39^ and Wang et al. found positive associations between tumor stage/grade and RDW, showing the utility of this parameter for predicting advanced carcinoma.^40^

In this study, ROC curve analysis showed that the optimal cutoff values of NLR, PLR, and RDW for predicting PSM after PCa were 2.31, 115.40, and 12.85%, respectively. Together, the three biomarkers yielded a greater AUC value, suggesting that it may be preferable to combine these indicators to improve the prediction of postoperative PSM. The result that the AUC of the three markers combined was 0.780 was validated by another group of patients, which was similar with training set. In the PSM group, NLR was positively correlated with PLR and RDW, and PLR was also positively correlated with RDW. In the NSM group, the only positive association was between NLR and PLR. It has been suggested that the higher the tumor risk, the stronger the interaction between NLR, PLR, and RDW and the higher the incidence of PSM. Importantly, values for NLR, PLR, and RDW can be obtained from routine blood tests in daily clinical practice, which is convenient, reproducible, and low cost. Numerous studies have confirmed that NLR, PLR, and RDW have better predictive value for patient prognosis in different cancers.^41^, ^42^, ^43^, ^44^ For example, Gu et al. demonstrated that elevated NLR was associated with poor overall survival (OS) and progression‐free survival (PFS)/recurrence‐free survival (RFS) in 16 266 patients with PCa.^45^ Similarly, a meta‐analysis conducted by Li et al. reported that in urological cancers (except for bladder cancer), elevated PLR was negatively related to OS.^46^ In addition, in a study conducted by Guan et al. on 3144 metastatic castration‐resistant prostate cancer (mCRPC) patients, NLR and PLR effectively predicted prognosis; these markers also served as indicators of the efficacy of personalized mCRPC treatment using abiraterone or enzalutamide.^47^ Interestingly, Fukuokaya et al. confirmed that high pretherapeutic RDW levels were significantly associated with worse PSA response and shorter PFS and OS in CRPC patients.^48^ Conversely, although RDW was found to be significantly related to tumor tissue size, stage, and necrosis in the study of Lee et al., no association was found with prognosis.^49^

Based on our results, intraoperative guidance including fluorescence and artificial intelligence can be used as an additional tool for patients with high NLR, PLR, or RDW before operation to improve outcomes. The guidance of augmented reality (AR) has mainly been used as a navigation system intraoperatively, and studies show a potential use of AR for more accurately identifying tumor margins and accuracy of detection of capsular involvement.^50^ Multiple preclinical and clinical studies have shown the usefulness of indocyanine green fluorescence in identifying and guiding treatment for PCa such as detecting surgical margin and pelvic lymph nodes, allowing a more accurate local staging and a prolonged biochemical RFS.^51^ The assessment of shaved prostate margins using fluorescence confocal microscopy (FCM) holds great potential as a valuable tool for secondary resection of spared neurovascular bundles in the case of PSM, aiming to maximize the preservation of functional tissue while pursuing oncological safety.^52^ The integrative approach including preoperative evaluation of clinicopathological features, intraoperative guidance of surgical margin, and postoperative follow‐up can benefit patients with PCa during the process of treatment.

Although our study is the first to explore the use of inflammatory parameters to predict postoperative PSM, several limitations should be acknowledged. First, this was a retrospective and single‐center study that involved a relatively small sample size. Hence, large‐scale prospective studies are needed to confirm the results and exclude biases from unknown confounders. Additionally, the LRPs were not conducted by the same urologist, which may have contributed to bias and the high incidence of PSM. Moreover, NLR, PLR, and RDW could be influenced by other conditions, such as acute coronary syndromes, valvular heart diseases, and renal conditions, as well as liver diseases, inflammatory diseases, and some medications.^53^ However, we did not take these factors into account in this study. Finally, this study only included Chinese patients, so the findings cannot be generalized to other ethnic groups.

## 5 CONCLUSION

Higher preoperative NLR, PLR, and RDW levels were significantly associated with postoperative PSM in PCa patients. Combining the three tests increased their capacity to predict PSM, which could potentially be beneficial for individualized predictions. Larger sample sizes with more varied ethnic backgrounds are urgently needed in the future to investigate the role of inflammatory parameters in predicting PSM.

## AUTHOR CONTRIBUTIONS

**Hao Wang:** Data curation (equal); writing – original draft (equal). **Dawei Xie:** Writing – original draft (equal). **Siqi Wang:** Writing – original draft (equal). **Liyang Wu:** Methodology (equal). **Yifan Chu:** Data curation (equal). **Pushen Yang:** Data curation (equal); resources (equal). **Weifeng He:** Resources (equal); software (equal). **Jianwen Wang:** Supervision (equal); writing – review and editing (equal).

## FUNDING INFORMATION

This work was supported by The Capital Health Research and Development of Special Fund, Beijing, China; grant number: 2020‐2‐2033.

## CONFLICT OF INTEREST STATEMENT

The authors declare that the research was conducted in the absence of any commercial or financial relationships that could be construed as a potential conflict of interest.

## ETHICS STATEMENT

The study was conducted in accordance with the Declaration of Helsinki and was approved by the ethics committee of Beijing Chaoyang Hospital, China (2020‐science‐299‐1).

## CONSENT TO PARTICIPATE

Beijing Chaoyang Hospital, China and got the exemption for informed consent.

## CONSENT FOR PUBLICATION

All participants in this study were exempted from informed consent.

## Supporting information


**Supplementary Table S1.** Clinical and pathological characteristics of PCa patients received LRP between NSM and PSM groups in validation set.

## Data Availability

The datasets used and/or analyzed during the current study are available from the corresponding author on reasonable request.
