# Supplementary material for: The value of preoperative neutrophil‐to‐lymphocyte ratio, platelet‐to‐lymphocyte ratio, and red blood cell distribution width in predicting positive surgical margin after laparoscopic radical prostatectomy
Source: Cancer Rep (Hoboken). 2024 Jan 23;7(2):e1977. doi: 10.1002/cnr2.1977 (PMC10849998; doi:10.1002/cnr2.1977)
Supplement: Supplementary file 1 — Supplementary Table S1. Clinical and pathological characteristics of PCa patients received LRP between NSM and PSM groups in validation set. [file CNR2-7-e1977-s001.docx]

**Supplementary Table Clinical and pathological characteristics of PCa patients received LRP between NSM and PSM groups in validation set.**

| Characteristics | n=95(%) | NSM group (n=35) | PSM group  (n=60) | t/χ^2^ | *P* value |
| --- | --- | --- | --- | --- | --- |
| Age(n,%), years |  |  |  | 2.954 | 0.086 |
| ≤65 | 28(29.5) | 14(40.0) | 14(23.3) |  |  |
| ＞65 | 67(70.5) | 21(60.0) | 46(76.7) |  |  |
| BMI(n,%), kg/m^2^ |  |  |  | 0.142 | 0.707 |
| BMI＜24.0 | 33(34.7) | 13(37.1) | 20(33.3) |  |  |
| BMI≥24.0 | 62(65.3) | 22(62.9) | 40(66.7) |  |  |
| Preoperative maximum PSA(n,%), ng/mL |  |  |  | 0.556 | 0.757 |
| ＜10 | 43(45.3) | 16(45.7) | 27(45.0) |  |  |
| 10~20 | 35(36.8) | 14(40.0) | 21(35.0) |  |  |
| ＞20 | 17(17.9) | 5(14.3) | 12(20.0) |  |  |
| PV(n,%), cm^3^ |  |  |  | 0.163 | 0.922 |
| ＜40 | 59(62.1) | 21(60.0) | 38(63.3) |  |  |
| 40~70 | 29(30.5) | 11(31.4) | 18(30.0) |  |  |
| ＞70 | 7(7.4) | 3(8.6) | 4(6.7) |  |  |
| f/t PSA(‾x±SD) | 95(100) | 0.12±0.06 | 0.14±0.05 | 1.346 | 0.181 |
| PSAD(‾x±SD), ng/mL^2^ | 95(100) | 0.45±0.54 | 0.50±0.47 | 0.437 | 0.663 |
| BPC ratio(‾x±SD) | 95(100) | 0.37±0.19 | 0.47±0.25 | 2.064 | 0.042* |
| NLR | 95(100) | 1.73±0.45 | 2.30±0.83 | 3.728 | 0.000* |
| PLR | 95(100) | 99.64±27.90 | 122.35±41.98 | 2.851 | 0.005* |
| RDW(%) | 95(100) | 12.52±0.70 | 13.10±0.80 | 3.563 | 0.001* |
| cT stage (n, %) |  |  |  | 5.388 | 0.020* |
| T2 | 56(58.9) | 26(74.3) | 30(50.0) |  |  |
| T3~T4 | 39(41.1) | 9(25.7) | 30(50.0) |  |  |
| D'Amico classification(n, %) |  |  |  | 1.479 | 0.477 |
| Low | 21(22.1) | 9(25.8) | 12(20.0) |  |  |
| Intermediate | 31(32.6) | 13(37.1) | 18(30.0) |  |  |
| High | 43(45.3) | 13(37.1) | 30(50.0) |  |  |
| Biopsy GS(n, %) |  |  |  | 0.768 | 0.681 |
| ≤6 | 25(26.3) | 11(31.4) | 14(23.3) |  |  |
| 7 | 30(31.6) | 10(28.6) | 20(33.3) |  |  |
| ≥8 | 40(42.1) | 14(40.0) | 26(43.4) |  |  |
| Pathological GS(n, %) |  |  |  | 6.856 | 0.032* |
| ≤6 | 7(7.4) | 3(8.6) | 4(6.7) |  |  |
| 7 | 50(52.6) | 24(68.6) | 26(43.3) |  |  |
| ≥8 | 38(40.0) | 8(22.8) | 30(50.0) |  |  |
| Prostate capsule invasion(n, %) |  |  |  | 0.510 | 0.475 |
| Yes | 20(21.1) | 6(17.1) | 14(23.3) |  |  |
| No | 75(78.9) | 29(82.2) | 46(76.7) |  |  |
| seminal vesicle invasion(n, %) |  |  |  | 4.284 | 0.038* |
| Yes | 22(23.2) | 4(11.4) | 18(30.0) |  |  |
| No | 73(76.8) | 31(88.6) | 42(70.0) |  |  |
| Pelvic lymph nodes involvement(n, %) |  |  |  | 2.204 | 0.138 |
| Yes | 8(8.4) | 1(2.9) | 7(11.7) |  |  |
| No | 40(42.1) | 16(45.7) | 24(40.0) |  |  |

* means the p-value<0.05 is considered statistically significant. Abbreviation:PSM, positive surgical margins; NSM, negative surgical margins; BMI, body mass index; PSA, prostate specific antigen; PV, prostate volume; PSAD, prostate specific antigen density; BPC, biopsy positive cores; NLR, neutrophil to lymphoctye ratio; PLR, platelte to lymphocyte ratio; RDW, red blood cell distribution width; cT stage, clinical T stage; GS, Gleason score.
